# Protein Kinase D Plays a Crucial Role in Maintaining Cardiac Homeostasis by Regulating Post-Translational Modifications of Myofilament Proteins

**DOI:** 10.3390/ijms25052790

**Published:** 2024-02-28

**Authors:** Melissa Herwig, Merima Begovic, Heidi Budde, Simin Delalat, Saltanat Zhazykbayeva, Marcel Sieme, Luca Schneider, Kornelia Jaquet, Andreas Mügge, Ibrahim Akin, Ibrahim El-Battrawy, Jens Fielitz, Nazha Hamdani

**Affiliations:** 1 Department of Cellular and Translational Physiology, Institute of Physiology, Ruhr University Bochum, 44801 Bochum, Germany; melissa.herwig@rub.de (M.H.); merima.begovic@rub.de (M.B.); heidi.budde@rub.de (H.B.); simin.delalat@rub.de (S.D.); saltanat.zhazykbayeva@rub.de (S.Z.); marcel.sieme@rub.de (M.S.); luca.schneider@rub.de (L.S.); kornelia.jaquet@rub.de (K.J.); ibrahim.el-battrawy@rub.de (I.E.-B.); 2 Institut für Forschung und Lehre (IFL), Molecular and Experimental Cardiology, Ruhr University Bochum, 44791 Bochum, Germany; andreas.muegge@rub.de; 3 Department of Cardiology, St. Josef-Hospital, UK RUB, Ruhr University Bochum, 44791 Bochum, Germany; 4 Department of Cardiology and Angiology, Bergmannsheil University Hospitals, UK RUB, Ruhr University Bochum, 44789 Bochum, Germany; 5 Department of Cardiology, Angiology, Haemostaseology and Medical Intensive Care, University Medical Center Mannheim, Medical Faculty Mannheim, Heidelberg University, 68167 Mannheim, Germany; ibrahim.akin@umm.de; 6 Department of Molecular Cardiology, DZHK (German Center for Cardiovascular Research), Partner Site, 17475 Greifswald, Germany; jens.fielitz@med.uni-greifswald.de; 7 Department of Internal Medicine B, Cardiology, University Medicine Greifswald, 17475 Greifswald, Germany; 8 Department of Physiology, University Maastricht, 6211 LK Maastricht, The Netherlands; 9 HCEMM-SU Cardiovascular Comorbidities Research Group, Department of Pharmacology and Pharmacotherapy, Semmelweis University, 1089 Budapest, Hungary

**Keywords:** protein kinase D, protein oxidation, myofilament proteins, inflammation, autophagy

## Abstract

Protein kinase D (PKD) enzymes play important roles in regulating myocardial contraction, hypertrophy, and remodeling. One of the proteins phosphorylated by PKD is titin, which is involved in myofilament function. In this study, we aimed to investigate the role of PKD in cardiomyocyte function under conditions of oxidative stress. To do this, we used mice with a cardiomyocyte-specific knock-out of Prkd1, which encodes PKD1 (Prkd1^loxP/loxP^; ^αMHC-Cre^; PKD1 cKO), as well as wild type littermate controls (Prkd1^loxP/loxP^; WT). We isolated permeabilized cardiomyocytes from PKD1 cKO mice and found that they exhibited increased passive stiffness (F_passive_), which was associated with increased oxidation of titin, but showed no change in titin ubiquitination. Additionally, the PKD1 cKO mice showed increased myofilament calcium (Ca^2+^) sensitivity (pCa_50_) and reduced maximum Ca^2+^-activated tension. These changes were accompanied by increased oxidation and reduced phosphorylation of the small myofilament protein cardiac myosin binding protein C (cMyBPC), as well as altered phosphorylation levels at different phosphosites in troponin I (TnI). The increased F_passive_ and pCa_50_, and the reduced maximum Ca^2+^-activated tension were reversed when we treated the isolated permeabilized cardiomyocytes with reduced glutathione (GSH). This indicated that myofilament protein oxidation contributes to cardiomyocyte dysfunction. Furthermore, the PKD1 cKO mice exhibited increased oxidative stress and increased expression of pro-inflammatory markers interleukin (IL)-6, IL-18, and tumor necrosis factor alpha (TNF-α). Both oxidative stress and inflammation contributed to an increase in microtubule-associated protein 1 light chain 3 (LC3)-II levels and heat shock response by inhibiting the mammalian target of rapamycin (mTOR) in the PKD1 cKO mouse myocytes. These findings revealed a previously unknown role for PKD1 in regulating diastolic passive properties, myofilament Ca^2+^ sensitivity, and maximum Ca^2+^-activated tension under conditions of oxidative stress. Finally, we emphasized the importance of PKD1 in maintaining the balance of oxidative stress and inflammation in the context of autophagy, as well as cardiomyocyte function.

## 1. Introduction

Protein kinase D (PKD) is a family of serine/threonine kinases that consist of three isoforms: PKD1, PKD2, and PKD3. All three isoforms are expressed in the heart, but the main isoform is PKD1 [1]. PKDs play crucial roles in regulating various processes in the cardiovascular system, including myocardial contraction, gene expression, cell survival metabolism, hypertrophy, and remodeling processes [2,3,4,5]. Numerous studies have suggested that PKD is involved in the development and progression of heart failure (HF). In fact, PKD1 expression has been found to be upregulated in a rabbit model of HF and in patients with dilated, ischemic, and hypertrophic cardiomyopathy [6,7]. PKD1 has been shown to regulate myocardial contraction by phosphorylating sarcomeric regulatory proteins such as myosin binding protein C (MyBPC) and cardiac troponin I (cTnI) [4,8]. For example, PKD-mediated phosphorylation of cMyBPC at Ser 302 may accelerate crossbridge kinetics [9]. PKD activity, as evidenced by PKD and MyBPC phosphorylation, has been shown to increase with increasing contraction frequency [10]. Cardiac troponin, which consists of the cardiac tropomyosin binding subunit (cTnT), the calcium (Ca^2+^)-binding subunit cTnC, and the inhibitory subunit cTnI, is responsible for the Ca^2+^-mediated interaction between myosin and actin. Phosphorylation of cTnI at Ser 23/24 by protein kinase A (PKA) promotes relaxation of myocytes by reducing the Ca^2+^ sensitivity of the myosin-actin interaction. Both serine residues in cTnI are also targets of PKD, and their PKD-dependent phosphorylation is associated with reduced myofilament Ca^2+^ sensitivity [5,6]. Recent studies have shown that in addition to sarcomeric proteins, the giant protein titin can also be phosphorylated by PKD1, resulting in a reduction in cardiomyocyte passive stiffness (F_passive_) [7]. The canonical model for PKD activation in cells involves receptor-dependent pathways that lead to the formation of diacylglycerol (DAG), which recruits PKD and activates protein kinase C (PKC). PKC phosphorylates PKD in its catalytic loop, releasing its autoinhibition and enabling autophosphorylation at several sites [11,12]. In vitro, DAG can directly activate PKD [12]. A PKC-independent activation of PKD via autophosphorylation is also possible in vitro [13,14]. In addition to diverse activation modes, several stimuli, such as G-protein coupled receptor agonists, cytokines, or growth factors, can activate PKD. Oxidative stress may also induce PKD activation, probably via src and abl tyrosine kinase signalling [15]. Oxidative stress occurs when the balance between the production of reactive oxygen species (ROS) and the cell’s ability to detoxify or eliminate these species is disrupted. It can cause severe damage to cellular components, leading to necrotic cell death, apoptosis, inflammation, and the progression of age-related diseases [6,16]. In HF, oxidative stress is thought to play a significant role in disease progression, as ROS can directly impair contractile function by oxidizing myofilament proteins or indirectly by modulating intracellular signaling pathways. Disulfide bonding decreases the extensibility of the cardiac-specific N2-B-unique sequence of titin, resulting in elevated cardiac stiffness [17]. Another study showed that S-glutathionylation of cryptic cysteines in the immunoglobulin (Ig) domains of the I-band region of titin decreases their mechanical stability and reduces the F_passive_ of human cardiomyocytes [18]. In a hypertensive mouse model, S-glutathionylation of cMyBPC has been associated with enhanced myofilament Ca^2+^ sensitivity and diastolic dysfunction [19]. Previous studies have also demonstrated that increased glutathionylation of cTnI, cMyBPC, and N2B-titin in end-stage human failing hearts is associated with altered maximum Ca^2+^-activated tension and Ca^2+^ sensitivity [20]. Inflammation, which accelerates heart disease, is characterized by an increase in pro-inflammatory cytokines and an imbalance between pro- and anti-inflammatory cytokines. Autophagy plays a vital role in maintaining cardiac homeostasis and function by regulating the production of ROS, inflammatory cytokines, and the removal of damaged and dysfunctional cells. Impaired autophagy contributes to the development of HF. Eisenberger-Lerner et al. have demonstrated that PKD acts as an effector of autophagy under oxidative stress conditions and is recruited to microtubule-associated protein 1 light chain 3 (LC3)-positive autophagosomes [21]. LC3 leads to the autophagy-mediated degradation of sequestome-1 or p62. Additionally, PKD has been shown to activate nuclear factor κ-light-chain-enhancer of activated B cells (NF-κB) in response to oxidative stress [15]. The aim of the current study was to investigate the role of PKD1 on cardiomyocyte function in response to oxidative stress. The hypothesis was that PKD1 knockout (KO) induces oxidative stress in cardiomyocytes. Therefore, myofilament Ca^2+^ sensitivity and cardiomyocyte max tension, as well as the phosphorylation and oxidation of sarcomeric proteins, inflammation, stress, and autophagy markers, were measured in cardiac tissue of cardiomyocyte-specific Prkd1 knockout (cKO) mice and wildtype (WT) littermate controls.

## 2. Results

### 2.1. Altered Maximum Ca^2+^-Activated Tension and Ca^2+^ Sensitivity Are Associated with Changes in cMyBPC Oxidation and Phosphorylation in PKD1 cKO Cardiomyocytes

**Figure 1:**
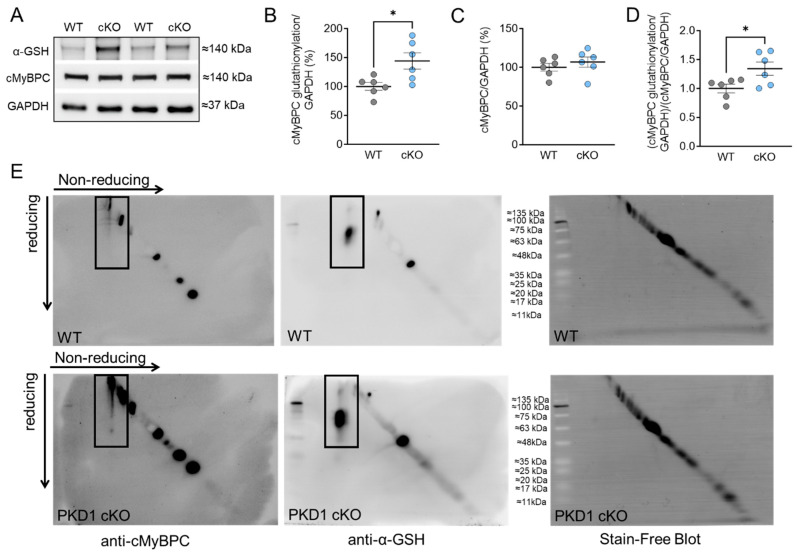
Cardiac myosin binding protein C (cMyBPC) oxidation in PKD1 cKO and WT cardiomyocytes. (**A**) Representative Western blots of cMyBPC-oxidation, cMyBPC-protein content and loading control GAPDH. Ratios of (**B**) glutathionylated cMyBPC over GAPDH, (**C**) cMyBPC-protein content over GAPDH and (**D**) glutathionylated over total cMYBPC (both normalized to GAPDH). Data are shown as mean ± SEM; *n* = 6 samples/group. * *p* < 0.05 PKD1 cKO vs. WT via unpaired Student’s *t*-test. (**E**) Representative diagonal gel electrophoresis of the WT (top panel) and PKD1 cKO (bottom panel). The non-reduced and reduced dimensions are shown in the image, with the molecular weight of the protein size standard in the center. Samples were initially run under non-reducing conditions in the first dimension and under reducing conditions in the second dimension. Blots were incubated with anti-cMyBPC antibody (left panel) and anti-glutathione (α-GSH; center panel). The stain-free blots are shown in the right panel. The black boxes indicate the oxidation of cMyBPC (left panel) and the glutathionylation (center panel), respectively.

**Figure 2:**
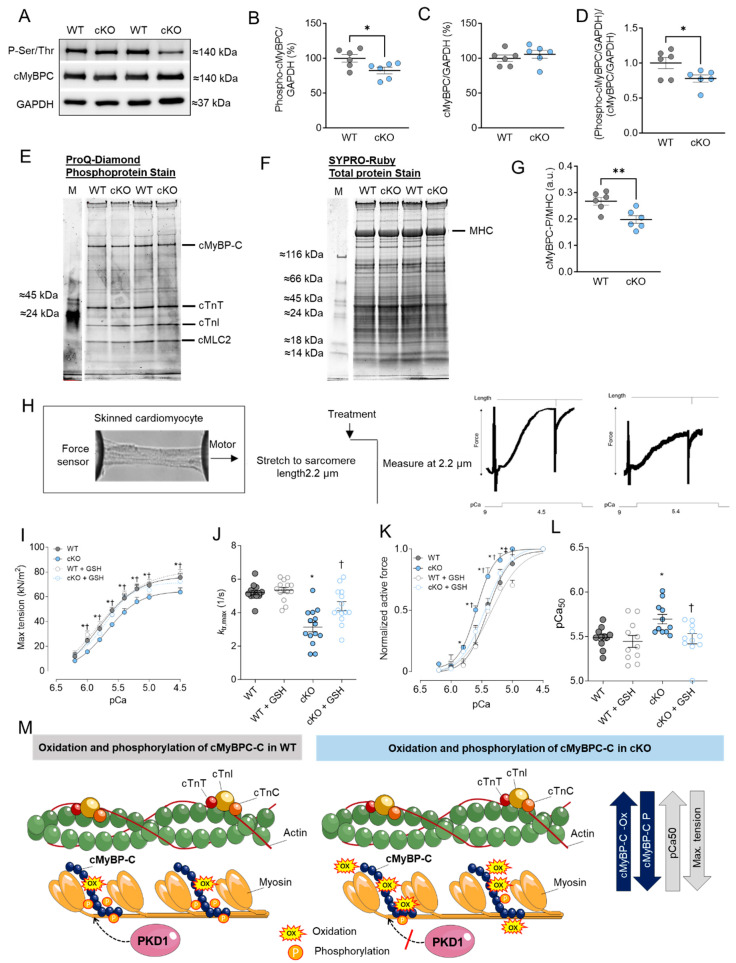
cMyBPC phosphorylation, cardiomyocyte max tension and calcium (Ca^2+^) sensitivity in PKD1 cKO and WT cardiomyocytes. (**A**) Representative Western blots of cMyBPC-phosphorylation, cMyBPC-protein content and loading control GAPDH. Ratios of (**B**) phosphorylated cMyBPC over GAPDH, (**C**) cMyBPC-protein content over GAPDH and (**D**) phosphorylated over total cMyBPC (both normalized to GAPDH). (**E**) ProQ-Diamond phosphoprotein stain, (**F**) SYPRO Ruby total protein stain and (**G**) ratio of phosphorylated cMyBPC over myosin heavy chain (MHC). (**H**) Representative image of skinned cardiomyocytes and stretch protocol. (**I**) Maximum tension of PKD1 cKO and WT cardiomyocytes before and after in vitro treatment with reduced glutathione (GSH) at different Ca^2+^ concentrations. (**J**) Cross-bridge cycling kinetics (ktr) at saturating [Ca^2+^] (at pCa 4.5; ktr, max) before and after in vitro GSH treatment. (**K**) Ca^2+^ sensitivity of PKD1 cKO and WT cardiomyocytes before and after in vitro GSH treatment different [Ca^2+^]. (**L**) pCa value for the half-maximal Ca^2+^-induced contraction before and after in vitro GSH. Data are shown as mean ± SEM; *n* = 6 samples/group. * *p* < 0.05 and ** *p* < 0.01 PKD1 cKO vs. WT via unpaired Student’s *t*-test. Panels (**I**–**L**): data are shown as mean ± SEM, (*n* = 16–20/4 cardiomyocytes/heart). * *p* < 0.05 PKD1cKO vs. WT, † *p* < 0.05 PKD1 cKO before vs. after GSH and ‡ *p* < 0.05 WT before vs. after GSH via one-way ANOVA. *p*-values were corrected for multiple comparisons via the Tukey method. (**M**) Scheme summarising the observed results in relation to the oxidation and phosphorylation of cMyBPC in WT and PKD1 cKO.

To investigate the impact of oxidative stress on the myofilament level in the hearts of PKD1 cKO animals, we initially measured the total oxidation (S-glutathionylation; Figure 1) and phosphorylation status (Figure 2) of cMyBPC, a crucial regulatory protein in myocardial contraction. S-glutathionylation of cMyBPC was assessed using an anti-glutathione antibody as described by Jeong et al. and Utter et al. [22,23] and the molecular weight of the glutathione-signal was verified with the assistance of the specific cMyBPC antibody. The PKD1 cKO hearts exhibited a significant increase in total S-glutathionylation compared to the WT hearts (Figure 1A,B), while the total cMyBPC content remained unchanged (Figure 1C). Consequently, the ratio of glutathionylation to total cMyBPC was higher in the PKD1 cKO hearts than in the WT hearts (Figure 1D). In addition, we also utilized diagonal gel electrophoresis to investigate and confirm the oxidation of cMyBPC. This technique allowed us to detect the formation of protein disulfides performing sequential non-reducing/reducing electrophoresis [24,25,26,27]. Proteins that did not form disulfides migrated identically in both dimensions and formed a diagonal after the second dimension. On the other hand, proteins containing intra-chain disulfides laid above the diagonal, while those that formed inter-chain disulfides fell below it diagonally [24,25,26,27]. We stained the samples with an anti-cMyBPC antibody (Figure 1E, left panel) and observed changes in the oxidation status of cMyBPC in the PKD1 cKO mouse heart (Figure 1E, bottom left panel) compared to WT (Figure 1E, top left panel), indicating the presence of disulfide bonds and the formation of homodimers through intramolecular disulfide bonding. No disulfide bond formation was detected in the WT (Figure 1E, top left panel), whereas a disulfide linkage at around 60 kDa was detected in the PKD1 cKO (Figure 1E, bottom left panel). Furthermore, we observed more glutathionylation in the PKD1 cKO than in the WT (Figure 1E, center panel).

Previously, we observed numerous phosphorylation sites in cMyBPC that were down-regulated in the PKD1 cKO compared to WT using in vivo quantitative mass spectrometric (MS) analysis [7]. Using Western blotting and a Phospho-Ser/Thr antibody, we detected a band around 140 kDa, which was presumably assigned, in addition to other proteins at this level, to the myofilament protein cMyBPC (Figure 2A). Phosphorylation status was notably lower in the PKD1 cKO hearts compared to the WT hearts (Figure 2A,B,M). The molecular weight of the phospho-signal was also checked using the specific cMyBPC antibody, which showed no alterations in the protein content level (Figure 2C). Similarly, the ratio of myofilament phosphorylation to its content exhibited a down-regulation in the PKD1 cKO mice compared to the WT (Figure 2D). In addition, we confirmed the hypo-phosphorylation of cMyBPC via ProQ-Diamond (phosphoprotein; Figure 2E) and SYPRO-Ruby (total protein; Figure 2F) staining. The ratio of phosphorylated cMyBPC over myosin heavy chain (MHC) was significantly reduced in the PKD1 cKO hearts compared to the WT hearts (Figure 2G).

Next, we examined the mechanical effects associated with the changes in cMyBPC oxidation and phosphorylation in PKD1 cKO cardiomyocytes. For this purpose, we measured Ca^2+^ sensitivity and the maximum tension of cardiomyocytes in a single-skinned state before and after treatment with reduced glutathione (GSH) at varying Ca^2+^ concentrations. The stretch protocol is depicted in Figure 2H. The maximum Ca^2+^-activated tension of single-skinned cardiomyocytes from PKD1 cKO hearts was significantly lower than that of WT mice (Figure 2I). To assess the ability to restore the reduced maximal tension, we conducted experiments with GSH treatment. Remarkably, maximum Ca^2+^-activated tension substantially increased in PKD1 cKO cardiomyocytes after GSH treatment, while this effect remained unchanged in cardiomyocytes from WT animals (Figure 2I). Additionally, the cross-bridge cycling kinetics (ktr) were significantly slower in the PKD1 cKO hearts compared to the WT, implying slower cross-bridge kinetics at saturating [Ca^2+^] (Figure 2J). In vitro GSH treatment could reverse this effect (Figure 2J).

The force-pCa relationship of single demembranated PKD1 cKO cardiomyocytes demonstrated a higher myofilament Ca^2+^ sensitivity (pCa_50_) when compared to the WT (Figure 2K). GSH treatment induced a rightward shift in the normalized force-pCa curve of the PKD1 cKO cardiomyocytes, indicating improved Ca^2+^ sensitivity following GSH treatment (Figure 2K). Notably, we observed a significantly higher pCa value for the half-maximal Ca^2+^-induced contraction (pCa_50_) in the PKD1 cKO mice compared to the WT group; GSH treatment reversed this increase, indicative of full recovery of myofilament function (Figure 2L). Our findings regarding the oxidation and phosphorylation of cMyBPC and their effects on pCa50 and maximum Ca^2+^-activated tension are summarized in Figure 2M.

### 2.2. Altered Troponin I (TnI) Phosphorylation Levels in PKD1 cKO Mice

**Figure 3:**
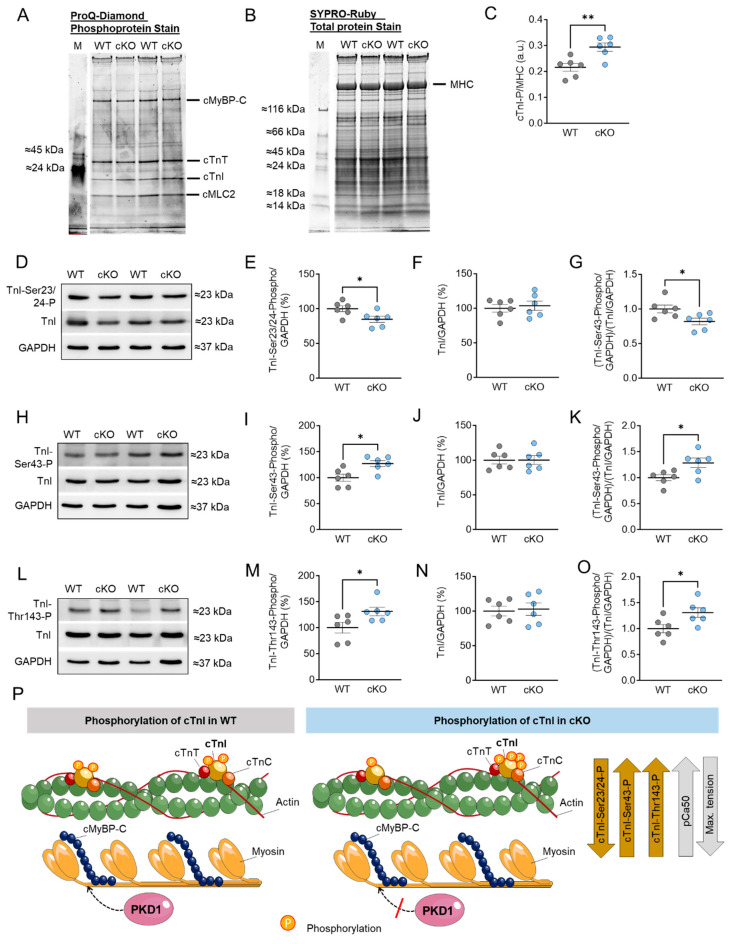
Cardiac troponin I (cTnI) phosphorylation in PKD1 cKO and WT hearts. (**A**) ProQ-Diamond phosphoprotein stain, (**B**) SYPRO Ruby total protein stain and (**C**) ratio of phosphorylated cTnI over myosin heavy chain (MHC). (**D**) Representative Western blots of TnI phosphorylation at Ser23/24, TnI-protein content and loading control GAPDH. Ratios of (**E**) TnI phosphorylation at Ser23/24 over GAPDH, (**F**) TnI-protein content over GAPDH and (**G**) TnI phosphorylation at Ser23/24 over TnI-protein content (both normalized to GAPDH). (**H**) Representative Western blots of TnI phosphorylation at Ser43, TnI-protein content and loading control GAPDH. Ratios of (**I**) TnI phosphorylation at Ser43 over GAPDH, (**J**) TnI-protein content over GAPDH and (**K**) TnI phosphorylation at Ser43 over TnI-protein content (both normalized to GAPDH). (**L**) Representative Western blots of TnI phosphorylation at Thr143, TnI-protein content and loading control GAPDH. Ratios of (**M**) TnI phosphorylation at Thr143 over GAPDH, (**N**) TnI-protein content over GAPDH and (**O**) TnI phosphorylation at Thr143 over TnI-protein content (both normalized to GAPDH). Data are shown as mean ± SEM; *n* = 6 samples/group. * *p* < 0.05 and ** *p* < 0.01 PKD1 cKO vs. WT via unpaired Student’s *t*-test. (**P**) Scheme summarising the observed results in relation to the phosphorylation of cTnI in WT and PKD1 cKO.

Increased Ca^2+^ sensitivity in PKD1 cKO mice (Figure 2I) was accompanied by increased total TnI phosphorylation assessed via ProQ-Diamond and SYPRO-Ruby staining (Figure 3A−C), reduced TnI phosphorylation at Ser23/Ser24 sites (Figure 3D,E,P), and increased phosphorylation at Ser43 (Figure 3H,I,P) and Thr143 (Figure 3L,M,P) sites in the PKD1 cKO mice compared to WT. The TnI content was unaltered between both groups (Figure 3F,J,N). The ratio of phosphorylated TnI to total TnI (both normalized to GAPDH) showed a significant reduction for the Ser23/Ser24 sites (Figure 3G), however, the phosphorylation levels of Ser43 (Figure 3K) and Thr143 (Figure 3O) were increased in the PKD1 cKO compared to WT. In contrast to cMyBPC, no S-glutathionylation was observed for TnI. Figure 3P illustrates the phosphorylation of cTnI and their effects on pCa_50_ and maximum Ca^2+^-activated tension.

### 2.3. Increased Cardiomyocyte Passive Stiffness (F_passive_) Is Accompanied by Increased S-Glutathionylation in PKD1 cKO Mice

**Figure 4:**
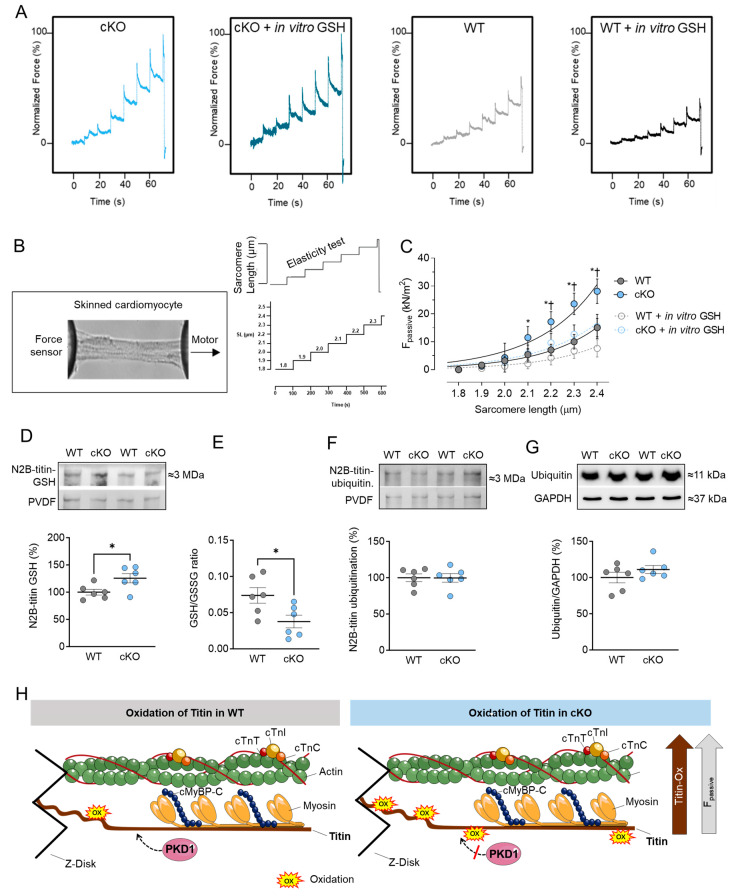
Cardiomyocyte passive stiffness (F_passive_) and titin glutathionylation and ubiquitination in PKD1 cKO and WT hearts. (**A**) Original recordings. (**B**) Stretch protocol of the force response to stepwise cell stretching of isolated skinned cardiomyocytes. (**C**) F_passive_ at sarcomere length 1.8–2.4 µm in the presence or absence of reduced glutathione (GSH). Curves are second-order polynomial fits to the means (±SEM; *n* = 16–20/4 cardiomyocytes/heart). * *p* < 0.05 PKD1 cKO vs. WT, † *p* < 0.05 PKD1 cKO before vs. after GSH via one-way ANOVA. (**D**) N2B-titin glutathionylation, (**E**) Ratio of reduced glutathione (GSH) over oxidized glutathione (GSSG), (**F**) N2B-titin ubiquitination and (**G**) ubiquitination levels in PKD1 cKO and WT hearts. Data are shown as mean ± SEM; *n* = 6 samples/group. * *p* < 0.05 PKD1 cKO vs. WT via unpaired Student’s *t*-test. (**H**) Scheme summarising the observed results in relation to the oxidation of titin in WT and PKD1 cKO.

Next to the Ca^2+^-activated tension and Ca^2+^ sensitivity, we measured the Ca^2+^-independent F_passive_ of cardiomyocytes within a sarcomere length (SL) range of 1.8 to 2.4 µm. Figure 4A,B show the elasticity test protocol and force recordings for both PKD1 cKO and WT cardiomyocytes. The passive SL-tension relationship of single skinned cardiomyocytes was generally steeper at SL beyond 2.1 µm in PKD1 cKO compared to WT cardiomyocytes (Figure 4C). In vitro treatment with GSH significantly reduced the cardiomyocyte F_passive_ at SL 2.1 to 2.4 µm in the PKD1 cKO mice, while no changes in the F_passive_ were observed after GSH treatment in the WT group (Figure 4C). One main contributor to myocardial F_passive_ is the giant protein titin, which is organized within the sarcomere and possesses elastic properties. Changes in titin isoform expression and post-translational modifications can impact myocardial elasticity and therefore influence the F_passive_ of cardiomyocytes. We assessed the oxidation (S-glutathionylation) and ubiquitination status of titin using Western blotting. The S-glutathionylation status of N2B-titin was significantly increased in PKD1 cKO compared to WT mice (Figure 4D,H). Consistent with this finding, the ratio of reduced to oxidized glutathione (GSH/GSSG ratio) was significantly reduced in PKD1 cKO compared to WT (Figure 4E). The N2B-titin ubiquitination and ubiquitin levels were unchanged between the two groups (Figure 4F,G).

### 2.4. Increased Inflammation Markers in PKD cKO Mice

**Figure 5:**
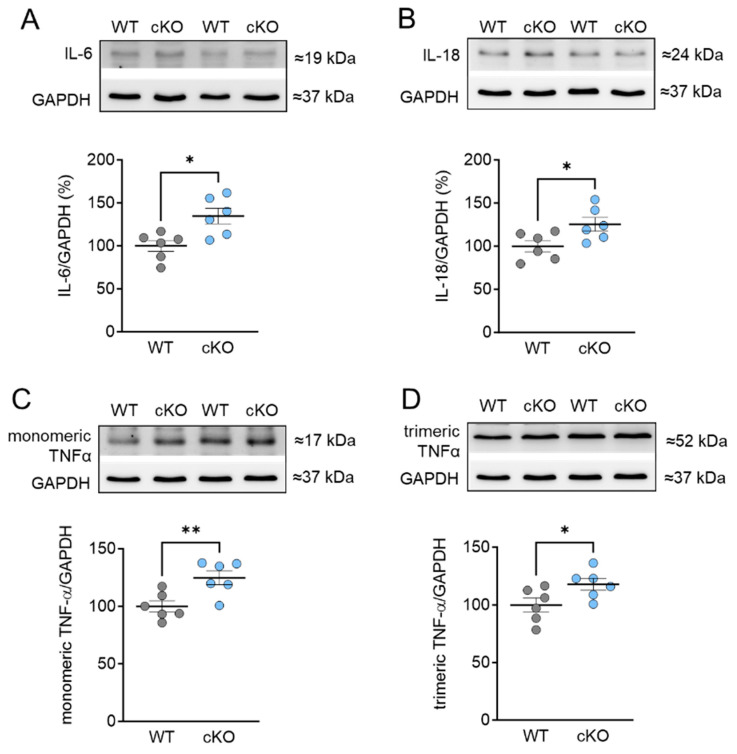
Expression of pro-inflammatory cytokines in PKD1 cKO and WT hearts. (**A**) Interleukin-6 (IL-6) protein level, (**B**) interleukin-18 (IL-18) protein level, (**C**) monomeric tumor necrosis factor alpha (TNF α) protein level and (**D**) trimeric TNF α protein level. Data are shown as mean ± SEM; *n* = 6 samples/group. * *p* < 0.05 and ** *p* < 0.01 PKD1 cKO vs. WT via unpaired Student’s *t*-test.

Based on our findings of oxidative stress-related changes in cMyBPC and titin, we proceeded to investigate the inflammatory responses associated with the cardiomyocyte-specific deletion of PKD1. To determine the expression of pro-inflammatory cytokines, including interleukin 6 (IL-6), 18 (IL-18), and tumor-necrosis factor alpha (TNFα), Western blot analysis was conducted. Our results revealed a significant increase in IL-6, IL-18, as well as both the monomeric and trimeric forms of TNFα in cKO hearts compared to WT hearts (Figure 5A–D). It is important to note that the trimeric form of TNFα is the biologically active form [28].

### 2.5. Stress Signaling and Autophagy Markers in PKD cKO Mice

**Figure 6:**
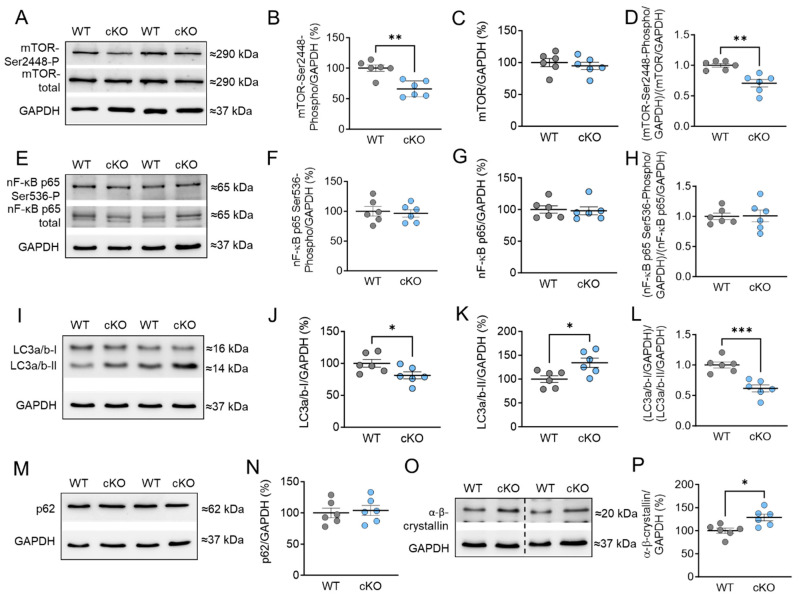
Markers of autophagy and stress signaling in PKD1 cKO and WT hearts. (**A**) Representative Western blots of mammalian target of rapamycin (mTOR)-phosphorylation at Ser2448, mTOR protein level and loading control GAPDH. Ratios of (**B**) phosphorylated mTOR over GAPDH, (**C**) mTOR-protein level over GAPDH, and (**D**) phosphorylated over total mTOR (both normalized to GAPDH). (**E**) Representative Western blots of nuclear factor kappa-light-chain-enhancer of activated B cells (NF-κB)-phosphorylation at Ser536, protein level and loading control GAPDH. Ratios of (**F**) phosphorylated NF-κB over GAPDH, (**G**) NF-κB-protein level over GAPDH, and (**H**) phosphorylated over total NF-κB (both normalized to GAPDH). (**I**) Representative Western blots of light chain 3 protein (LC3) forms I and II and loading control GAPDH. Ratios of (**J**) LC3-I over GAPDH, (**K**) LC3-II over GAPDH, and (**L**) LC3-I over LC3-II (both normalized to GAPDH). (**M**) Western blot of Sequestosome 1 (p62) marker and (**N**) p62 protein level. (**O**) Western blot of α-β-crystallin and (**P**) α-β-crystallin protein level. Data are shown as mean ± SEM; *n* = 6 samples/group. * *p* < 0.05, ** *p* < 0.01 and *** *p* < 0.001 PKD1 cKO vs. WT via unpaired Student’s *t*-test.

In addition, we examined the phosphorylation and protein content of mammalian target of rapamycin (mTOR), which plays a crucial role in regulating various signaling pathways related to cell homeostasis, apoptosis, autophagy, and cardiomyocyte growth. We observed that the phosphorylation of mTOR at Ser2448 was significantly reduced in PKD1 cKO mice compared to WT mice (Figure 6A,B). However, the total amount of mTOR was unchanged across the groups (Figure 6C). The ratio of phosphorylated mTOR to total mTOR was decreased in PKD1 cKO mice compared to WT mice (Figure 6D). We also examined nuclear factor kappa-light-chain-enhancer of activated B cells (NF-κB) p65, which is involved in the regulation of inflammatory and stress signaling pathways. The phosphorylation levels of NF-κB p65 at Ser536 and its protein content remained unaltered (Figure 6E–H).

Furthermore, we investigated other markers for autophagy, such as light chain 3 (LC3) and sequestosome-1 (p62). During autophagy, the cytosolic LC3-I form is conjugated with phosphatidylethanolamine to form the LC3-II form, indicating autophagosome formation [29]. We found that the protein content of cytosolic LC3-I was significantly decreased, while the LC3-II form was significantly increased in PKD1 cKO tissue compared to WT tissue (Figure 6I–K). The ratio of LC3-I to LC3-II showed a reduction in PKD1 cKO mice compared to WT mice (Figure 6L). However, the protein levels of p62, a ubiquitin-binding protein, were unchanged in both groups (Figure 6M,N). This suggests that the absence of PKD1 blocks the maturation of autophagosomes and their fusion with lysosomes. In addition, we conducted autophagic flux assays with Bafilomycin A1 (BafA1) (Supplementary Materials Figure S1A) ex vivo for 1 h in the myocardial tissue samples, which inhibits the vacuolar-type H^+^-ATPase at the lysosome and thus prevents the fusion of autophagosomes with lysosomes [30,31,32,33]. However, we did not observe any changes in LC3-II and p62 levels in PKD1 cKO and WT after in vitro treatment with BafA1 (Supplementary Materials Figure S1A–C). We determined the autophagic flux by dividing the protein levels of either LC3-II (Supplementary Materials Figure S1B) or p62 (Supplementary Materials Figure S1C) in BafA1-treated samples by those without the inhibitor. The results showed no changes in the autophagic flux for LC3-II (Supplementary Materials Figure S1D) and p62 (Supplementary Materials Figure S1E) between PKD1 cKO and WT. Additionally, we explored the involvement of heat shock proteins (HSPs) in maintaining proteostasis under stress. HSPs help in protein folding and prevent protein aggregation. We observed a significant increase in the protein content of HSP α-β-crystallin in PKD1 cKO mice compared to WT mice (Figure 6O,P).

## 3. Discussion

Here we demonstrated increased oxidation of cMyBPC and titin, indicating oxidative stress in cardiomyocytes of PKD1 cKO mice. The oxidation of myofilament proteins was associated with higher myofilament pCa_50_ and lower maximum Ca^2+^-activated tension, which were linked to reduced phosphorylation of cMyBPC and altered phosphorylation of TnI. Elevated cardiomyocyte F_passive_ was observed alongside increased titin oxidation, while titin ubiquitination remained unaffected. Treatment of permeabilized cardiomyocytes with the reducing reagent GSH improved the increased F_passive_ and pCa_50_ and significantly reduced maximum Ca^2+^-activated tension, providing strong support for the hypothesis that oxidative stress is increased in PKD1 cKO mice. Additionally, it suggests that myofilament protein oxidation contributes to cardiomyocyte dysfunction.

Signal transduction processes play a critical role in the physiology and pathophysiology of the heart. Protein kinases transfer phosphate groups from adenosine triphosphate to serine, threonine, or tyrosine residues on their target substrates, inducing a conformational change that leads to differences in the activity, localization, and function of many proteins [34]. Therefore, protein kinases are considered key regulators of cellular function, and multiplex kinase signaling has been shown to be an important modulator of cardiac function at the sarcomeric protein level [35]. A balance between phosphorylation and dephosphorylation events is crucial, as dysfunction of kinase activities and phosphorylation processes, as well as dysregulation of signaling pathways, are common in the pathogenesis of HF. The serine/threonine kinase PKD plays an essential role in the heart by modulating myofilament proteins involved in myocardial contraction, regulating the expression of hypertrophic genes, remodeling processes, and metabolism [2,3]. Oxidative stress and inflammation are important pathophysiological modulators in the development and progression of HF. Since PKD can be activated in response to oxidative stress [15], we aimed to investigate the impact of PKD1 on cardiomyocyte function in response to oxidative stress.

### 3.1. Role of PKD in Regulating Sarcomeric Thin and Thick Filament Function

PKD plays a crucial role in regulating the function of sarcomeric thin filaments. In vitro phosphorylation assays have shown that PKD mediates the phosphorylation of myocardial substrates such as cTnI and cMyBPC [8]. The major targets of PKD phosphorylation have been identified as the Ser22 and Ser23 sites in cTnI (referring to the human sequence; equivalent to Ser23 and Ser24 in mouse) [8,36]. These sites are also targeted by PKA and protein kinase G (PKG), resulting in a reduction in myofilamental Ca^2+^ sensitivity [37,38,39]. In fact, the PKD-mediated phosphorylation of cTnI in skinned myocytes from the adult rat left ventricle has been shown to reduce Ca^2+^ sensitivity, accompanied by a rightward shift of the tension-pCa relationship, while not altering maximal tension [8]. However, in our study, we observed an approximately 18% reduction in Ser23/24 phosphorylation and a significant decrease in maximum Ca^2+^-activated tension of single-skinned cardiomyocytes from PKD1 cKO hearts, along with an increase in pCa_50_ compared to WT mice. All of these changes are attributed to the loss of PKD1. The altered maximal tension suggests that other phosphorylation sites and myofilamental proteins are also contributing to these effects. Additionally, these data confirm our previous findings from in vivo quantitative MS analysis, which revealed a significant reduction in cTnI phosphorylation at the Ser23 and Ser24 sites [7].

Under normal physiological conditions, the phosphorylation status of the thick filament-associated protein cMyBPC is finely tuned, enabling the regulation of myocardial contraction and relaxation. Several studies have reported a deficit in cMyBPC phosphorylation, accompanied by impaired cardiac contractility in HF patients [40,41,42]. It has been demonstrated that PKD phosphorylates cMyBPC at Ser302 and Ser315, thereby accelerating cross-bridge cycle kinetics and increasing maximal Ca^2+^-activated contraction tension [9,10]. Our findings show a significant decrease in overall cMyBPC phosphorylation in PKD1 cKO mice, accompanied by a significant increase in S-glutathionylation. This opposite development of oxidative and phosphorylative modifications of myofilament proteins, especially cMyBPC, has been observed in several studies conducted by us and others. Stathopoulou et al. have shown an increase in S-glutathionylation of cMyBPC in HF patients [43]. They have also proposed that this may be the reason for the impaired phosphoregulation of cMyBPC and myofilamental kinetics [43]. Another study has demonstrated that in stressed rat hearts, cMyBPC S-glutathionylation is accompanied by reduced PKA-dependent phosphorylation of cMyBPC and cTnI, leading to increased myofilament Ca^2+^ sensitivity and reduced systolic and diastolic properties [44]. Our previous studies have shown that the altered mechanical properties of failing human cardiomyocytes are associated with increased S-glutathionylation and reduced phosphorylation of cMyBPC and cTnI, suggesting an interplay between these two post-translational modifications [20]. However, the causal relationship between phosphorylation and oxidative modifications and the extent to which phosphorylated or oxidatively modified myofilament proteins are involved in the progression of heart disease remain to be elucidated. Our results suggested that PKD1 is necessary for maintaining the balance of redox pathways associated with thick and thin filament function, as well as cardiomyocyte elasticity.

### 3.2. PKD and Titin–Role in Cardiomyocyte Function and Mechanics

Proper cardiomyocyte function and cardiac contractility partially rely on passive myocardial stiffness, which is mainly attributed to the giant protein titin [45]. The elasticity of titin in cardiomyocytes can be altered through both transcriptional and post-translational modifications, such as phosphorylation and oxidation [46]. In previous studies, we demonstrated that titin is a substrate of PKD1 by using cardiomyocyte-specific PKD1 knockout mice and in vivo quantitative MS analysis [7]. We identified several phosphosites along the entire titin molecule, and the specific deletion of PKD1 in cardiomyocytes was associated with an overall reduction in titin phosphorylation and an increase in phosphorylation at sites that are dependent on PKC and Ca^2+^/calmodulin-dependent protein kinase II (CaMKII) [7]. Cardiomyocyte F_passive_ was increased in cardiomyocytes from the PKD1 cKO mice, but this could be corrected via in vitro administration of PKD1 [7].

Our experiments confirmed the previous findings that PKD1-dependent phosphorylation decreases F_passive_. However, we have also observed that, in addition to reduced titin phosphorylation in PKD1 cKO [7], oxidation through S-glutathionylation also plays a significant role in the elevation of myocardial tension. The increased F_passive_, which indicates diastolic dysfunction, was accompanied by approximately a 25% increase in S-glutathionylation of titin. Consistent with previous research, we found high levels of oxidative stress in PKD1 cKO hearts, as evidenced by a reduced GSH/GSSG ratio, suggesting a connection between oxidative stress and increased myocardial stiffness. Treatment with GSH in vitro was able to restore the elevated F_passive_, demonstrating its antioxidant potential.

Titin can be modified under oxidizing conditions through the formation of disulfide bonds and S-glutathionylation of cryptic cysteines [17,18,47]. Studies have shown that disulfide bonding decreases the extensibility of the cardiac-specific N2-B-unique sequence of titin, resulting in increased F_passive_ [17]. Additionally, another study has demonstrated that S-glutathionylation of cryptic cysteines in the Ig domains of the I-band region reduces their mechanical stability and decreases cardiomyocyte F_passive_ [18].

### 3.3. Inflammation, Oxidative Stress, Stress Signaling, and PKD1

During oxidative stress conditions, PKD1 is activated through the production of mitochondrial DAG by phospholipase D activity. This activation is further enhanced by phosphorylation of two Tyr residues within the PKD1 molecule [48,49,50]. It has also been discovered that PKD1 is located in the mitochondria of cells exposed to both exogenous and mitochondrial ROS [51]. Once activated by ROS, PKD1 triggers the NF-κB pathway, which regulates the expression of superoxide dismutase 2 (SOD2) and aids in cellular detoxification [15,50,51]. However, our study did not observe any changes in either phosphorylation or the amount of NF-κB in the PKD1 cKO mice. Oxidative stress and inflammation are known to cause microvascular endothelial dysfunction, leading to cardiac hypertrophy and interstitial fibrosis in HFpEF [52]. The PKD1 cKO hearts exhibited high levels of pro-inflammatory cytokines IL-6, IL-18, and TNFα, which were associated with decreased GSH levels. All of these factors contributed to the observed changes in protein modifications, Ca^2+^ sensitivity, and F_passive_. Molecular chaperones such as α-ß-crystallin and HSP27, which is also a PKD substrate [53], are supposed to protect cardiomyocytes from damage by preventing stress-induced protein aggregation [54,55]. Hassoun et al. demonstrated that α-ß-crystallin can ameliorate the increased F_passive_ in HCM cardiomyocytes [56]. In our study, the levels of α-β-crystallin were increased in PKD1 cKO hearts. Previously, we have shown that in HCM hearts, increased PKD and HSP27 activity were associated with increased oxidative stress and the relocation of HSP27 away from the Z-disk and I-band region [7]. Our data suggested that α-ß-crystallin may also fail to exert its cytoprotective effects on titin extensibility, possibly due to oxidative modification and thus induction of translocation.

Another mechanism to maintain cellular homeostasis under stress conditions involves autophagy, a process by which cytosolic components such as organelles, proteins, and lipids are disposed of via lysosomal degradation pathways [57]. Studies suggest that autophagy occurs in response to compromised conditions such as oxidative stress, metabolic stress, inflammation, and cell death in the heart. In particular, autophagy is thought to play a role in the clearance of damaged proteins and organelles, thereby reducing oxidative stress and inflammation [58]. Additionally, autophagy may protect against cell death by promoting the clearance of damaged organelles and proteins. Therefore, autophagy may be an important target in the treatment of HF [59]. It has been shown that PKD acts as an effector of autophagy under oxidative stress conditions and is recruited to LC3-positive autophagosomes [21]. LC3 leads to the autophagy-mediated degradation of sequestome-1, also known as p62. In our study, we observed an increase in LC3-II expression, indicating the formation of autophagosomes in the absence of PKD1. The increase in LC3-II levels observed in PKD1 cKO hearts could indicate either active autophagic processes or a blockage of autophagy due to inefficient autophagosomal maturation and/or fusion with the lysosome [60,61]. However, the expression of p62 was unchanged in PKD1 cKO hearts compared to WT. Autophagic flux assays were conducted using Bafilomycin A1 (BafA1) ex vivo as an inhibitor of autophagosome fusion with lysosomes [30,31,32,33]. The results showed that the number of autophagosomes did not change and the levels of LC3-II in the BafA1-treated samples were similar to those in non-treated samples. The lack of effect could be attributed to several reasons such as compensatory mechanisms in response to inhibition of autophagy by BafA1. These mechanisms may activate alternative degradation pathways or modify signaling pathways, which could be particularly prevalent in our studies involving mice as experimental models.

Moreover, it is worth noting that previous studies have shown that the effects of BafA1 treatment in cell culture and in vivo require hours to manifest fully. Hence, the lack of observable effects in our experiments could be because the 1-h incubation time may not have been sufficient to induce significant changes. Nevertheless, the PKD1 cKO hearts were characterized by increased stress due to the accumulation of oxidative stress indicative of oxidative modifications of myofilament proteins, the increase in pro-inflammatory markers, higher myofilament pCa_50_, lower maximum Ca^2+^-activated tension, and increased F_passive_ in the PKD1 cKO mice, all of which contributed to the observed cardiomyocyte dysfunction. Furthermore, the PKD1 cKO mice attempted to maintain cellular homeostasis by activating autophagy, as evidenced by increased levels of LC3-II, but since p62 was unchanged, the maturation process of the autophagosome appeared to be impaired. mTOR serves as a key regulator of cell growth, autophagy, and survival. In response to cellular stress, a reduction in mTOR phosphorylation at Ser2448 can be observed, which in turn leads to the induction of autophagy [62]. The PKD1 cKO heart showed an increase in oxidative and inflammatory stress accompanied by reduced mTOR phosphorylation at Ser2448 and unchanged mTOR expression. Together with the increase in LC3-II expression, this showed the presence of autophagic processes upon removal of cardiac PKD1. Confirming our results, a study by Zhao et al. showed in a transverse aortic constriction-induced hypertrophy mouse model that PKD1 contributed to the development of cardiac hypertrophy by inhibiting AKT/mTOR-regulated cardiac autophagy [63].

## 4. Materials and Methods

### 4.1. Animal Model and Tissue Sampling

All animal experiments were performed in accordance with the guidelines of Charité Universitätsmedizin Berlin and Max-Delbrück Center for Molecular Medicine with the permission of Landesamt für Gesundheit und Soziales (LaGeSo, Berlin, Germany) for the use of laboratory animals (permit number: G 0229/11) and following the ‘Principles of Laboratory Animal care’ (NIH publication no. 86-23, revised 1985) as well as the current version of German Law on the Protection of Animals. The generation and usage of the conditional *Prkd1* allele was published in 2008 [5,64]. Cardiomyocyte specific *Prkd1* knock-out mice were generated by using the Cre-loxP recombination system by crossing *Prkd1*^loxP/loxP^ mice with Cre carrying mice controlled by cardiomyocyte-specific α-myosing-heavy-chain promotor (αMHC-Cre) [65] (cKO, *Prkd1*^loxP/loxP; αMHC-Cre^). αMHC-Cre-negative animals were used as controls (WT, *Prkd1*^loxP/loxP^). At 8–10 weeks of age, animals were sacrificed, hearts and left ventricles (LV) were quickly excised, snap frozen in liquid nitrogen, and stored at −80 °C until further use.

### 4.2. Force Measurements in Single Skinned Cardiomyocytes

Force measurements were performed on single de-membranated cardiomyocytes (*n* = 20–26/4 hearts/group) as described before [66]. Briefly, LV samples were de-frozen in relaxing solution (containing in mM: 1.0 free Mg^2+^; 100 KCl; 2.0 EGTA; 4.0 Mg-ATP; 10 imidazole; pH 7.0), mechanically disrupted and incubated for 5 min in relaxing solution supplemented with 0.5% Triton X−100 (all from Sigma-Aldrich, Saint Louis, MO, USA). The cell suspension was washed 5 times in relaxing solution. Single cardiomyocytes were selected under an inverted microscope (Zeiss Axiovert 135, 40× objective; Carl Zeiss AG Corp, Oberkochen, Germany) and attached with silicone adhesive between a force transducer and a high-speed length controller (piezoelectric motor) as part of a “Permeabilized Myocyte Test System” (1600A; with force transducer 403A; Aurora Scientific, Aurora, ON, Canada). Cardiomyocyte Ca^2+^-independent passive force (F_passive_) was measured in relaxing buffer at room temperature (RT) within a sarcomere length (SL) range between 1.8 and 2.4 μm. Force values were normalized to myocyte cross-sectional area calculated from the diameter of the cells, assuming a circular shape. F_passive_ was thereafter measured within a SL range between 1.8 and 2.4 μm as described above. Thereafter, the cardiomyocyte was adjusted to 2.2 μm SL and exposed to a series of solutions with different Ca^2+^ concentrations ranging from pCa 9.0 (relaxing) to pCa 4.5 (maximal activation) to obtain the force–pCa relation. Mean values on relative force (and tension) vs. pCa diagrams were fit with the “Hill” equation, resulting in a sigmoidal curve.

### 4.3. Protein Isolation and Western Blot Analysis

The amount and phosphorylation of proteins was determined using sodium dodecylsulfate polyacrylamide gel electrophoresis (SDS-PAGE) followed by Western blot analysis. Myocardial left ventricular (LV) tissue samples (*n* = 6 samples/group) were homogenized with modified Laemmli buffer (0.05 M Tris-HCl pH 6.8, 8 M urea, 2 M thiourea, 3% SDS (*w*/*v*), 0.03% ServaBlue (*w*/*v*), 10%/*v*/*v*) glycerol, 75 mM DTT). To study the glutathionylation of proteins, N-ethylmaleimide (NEM) instead of DTT was used in the modified Laemmli buffer. After 20 min incubation on ice, samples were heated for 3 min at 96 °C and centrifuged for 3 min at 14,000 rpm. The concentration of the samples was determined using the Pierce^TM^ 660 nm protein assay (Thermo Fisher Scientific, Waltham, MA, USA). A total of 15 µg of the sample supernatant was loaded and separated via electrophoresis using 8%, 10% or 15% SDS gels (depending on the molecular weight of the protein of interest) and run at 90 V for 20 min followed by 125 V for about 90 min. After SDS-PAGE, the gels were blotted onto polyvinylidene difluoride (PVDF) membranes (Immobilon-P 0.45 μm; Merck Millipore, Burlington, MA, USA). Blots were blocked with 5% bovine serum albumin (BSA) in Tris-buffered saline with Tween (TBST) for 1 h at RT and subsequently incubated with primary antibodies overnight at 4 °C (Table 1). GAPDH was used as loading control. After washing with TBST, primary antibodies were detected with HRP-conjugated secondary anti-rabbit or anti-mouse antibodies (Table 2) and enhanced chemiluminescence (Clarity Western ECL Substrate, BioRad, Munich, Germany). Imaging was carried out with a ChemiDoc Imaging system (BioRad). Stained protein bands were quantified via densitometry using the Multi Gauge V3.2 software (FUJIFILM Corp, Minato, Tokyo, Japan). Finally, the signals obtained for the amounts of total protein and phosphorylated protein were normalized to signals obtained from GAPDH stained membranes.

### 4.4. Titin Analysis

To analyze titin post-translational modification, LV tissues samples were homogenized in modified Laemmli buffer. Samples were heated at 96 °C for 3 min, centrifuged for 3 min at 4 °C at 14,000 rpm, and then separated via agarose strengthened 2% SDS-PAGE [7,67]. Gels were run at 2–4 mA constant current per gel for 16 h. Proteins were blotted on PVDF membranes (Immobilon-P 0.45 μm; Merck Millipore, Burlington, MA, USA). Blots were pre-incubated with 5% BSA in TBST for 1 h at RT followed by primary antibody incubation overnight at 4 °C. Titin ubiquitination was determined via an anti-ubiquitin antibody (Table 1) and glutathionylation of titin via an anti-GSH antibody (Table 1). After washing with TBST, primary antibodies were detected with HRP-conjugated secondary anti-rabbit or anti-mouse antibodies (Table 2) and enhanced chemiluminescence (Clarity Western ECL Substrate, BioRad, Munich, Germany). Chemiluminescence signals were normalized to signals obtained from Coomassie-stained PVDF membranes referring to the entire protein amount transferred. The results were quantitated via densitometry using Multi Gauge V3.2 software.

### 4.5. ProQ-Diamond and SYPRO-Ruby Staining

To analyze the phosphorylation status of the myofilament proteins cMyBPC and cTnI, we used the dual staining system ProQ-Diamond/SYPRO-Ruby (Thermo Fisher Scientific) according to manufacturers’ instructions. Homogenized LV samples were separated on 4–15% Criterion^TM^TGX^TM^ precast gels (BioRad, Munich, Germany) and stained with ProQ-Diamond for 1 h and subsequently with SYPRO Ruby overnight. Proteins were visualized using the ChemiDoc imaging system (BioRad). Stained protein bands were quantified via densitometry using the Multi Gauge V3.2 software (FUJIFILM Corp, Minato, Tokyo, Japan). Phospho-signals on ProQ-Diamond-stained gels were normalized to the corresponding myosin heavy chain (MHC) total protein signal on SYPRO-Ruby stained gels.

### 4.6. Diagonal Gel Electrophoresis

Homogenized LV tissues samples (with NEM) were first electrophoresed in a non-reducing SDS PAGE followed by treatment of the non-reduced gel (first dimension) with 50 mM NEM to reduce disulfide bonds. The reduced proteins were then electrophoresed in a second dimension in a reducing gel. Proteins were blotted onto PVDF membranes as written above. The blots were incubated with either cMyBPC or α-GSH antibodies (see Table 1).

### 4.7. Ex Vivo Autophagic Flux Assay Using Western Blot

To study autophagic flux, myocardial LV tissue samples (*n* = 6 samples/group) were incubated for 1 h at 37 °C in relaxing solution (containing in mM: 1.0 free Mg^2+^; 100 KCl; 2.0 EGTA; 4.0 Mg-ATP; 10 imidazole; pH 7.0; supplemented with 0.4% protease inhibitor cocktail (Sigma-Aldrich P8340)) with either 500 nM Bafilomycin A1 (Selleckchem S1413; BafA1) or vehicle (without BafA1). Samples were homogenized, separated via SDS-PAGE and blotted onto PVDF membranes as written above. The blots were incubated with either LC3a/b or SQSTM1/p62 (see Table 1). Autophagic flux ratio was calculated as BafA1/vehicle.

### 4.8. Quantification of Glutathione Level in Myocardial Homogenates

Total glutathione (GSH) levels in myocardial homogenates (*n* = 6 LV samples/group) were determined in triplicate using a colorimetric glutathione assay kit (CS0260, Sigma-Aldrich) according to manufacturer’s instructions.

### 4.9. Statistical Analysis

Data are given as mean values ± SEM. For statistical analysis of the two groups of parametric data Student’s *t*-test was used. For analysis of parametric data comparing more than two groups one-way ANOVA was used and *p*-values were corrected for multiple comparisons via the Tukey method. *p*-values < 0.05 were considered to reflect statistically significant differences. All analyses were performed using GraphPad Prism 10.

## 5. Conclusions

Our data indicates that PKD1 has a crucial role in balancing cellular oxidation/reduction, inflammation, and regulation of sarcomeric function in cardiomyocytes. If PKD1 is knocked out, oxidative stress occurs, which can lead to changes in diastolic passive properties, myofilament Ca^2+^ sensitivity, and maximum Ca^2+^-activated tension. These findings emphasize the significance of PKD1 in preserving the function of cardiomyocytes and maintaining cellular homeostasis.

## Figures and Tables

**Table 1:** List of primary antibodies.

Primary Antibody	Company	Cat. No.	Dilution
SQSTM1/p62	Cell Signaling Technology (Leiden, The Netherlands)	39749S	1:1000
LC3a/b	Cell Signaling Technology	12741S	1:1000
mTor-total	Cell Signaling Technology	2972S	1:1000
Phospho-mTor (Ser2448)	Cell Signaling Technology	2971S	1:1000
Ubiquitin	Cell Signaling Technology	43124S	1:1000
Phospho-Serine/Threonine antibody	ECM Bioscience LLC (Versailles, KY, USA)	PP2551	1:500
α-GSH	Abcam (Cambridge, UK)	ab19534	1:1000
IL-6	Invitrogen (Waltham, MA, USA)	P620	1:1000
IL-18	Invitrogen	PA5-80719	1:1000
TNF-α	Invitrogen	AMC3012	1:1000
NF-κB p65	Cell Signaling Technology	8242S	1:1000
Phospho-NF-κB p65 (S536)	Cell Signaling Technology	3033S	1:1000
Myosin-binding protein C	Invitrogen	PA571701	1:2000
Phospho-cardiac troponin I (Ser_23/24_) antibody	Cell Signaling Technology	4004S	1:1000
Phospho-cardiac troponin I (phospho Ser_43_) antibody	Abcam	ab196005	1:1000
Phospho-cardiac troponin I (phospho Thr_143_) antibody	Abcam	ab58546	1:1000
Troponin I	Cell Signaling Technology	4002S	1:1000
α-β-crystallin	Abcam	ab13497	1:1000
GAPDH	Cell Signaling Technology	97166	1:2000
GAPDH	Cell Signaling Technology	2118	1:2000

**Table 2:** List of secondary antibodies.

Secondary Antibody	Company	Cat. No.	Dilution
Anti-Rabbit	Cell Signaling Technology	7074	1:10,000
Anti-mouse	Cell Signaling Technology	7076	1:10,000

## Data Availability

The data presented in this study are available in the article and Supplementary Materials.

## Supplementary Materials

The supporting information can be downloaded at: https://www.mdpi.com/article/10.3390/ijms25052790/s1.
