# Supplementary material for: Protein Kinase D Plays a Crucial Role in Maintaining Cardiac Homeostasis by Regulating Post-Translational Modifications of Myofilament Proteins
Source: Int J Mol Sci. 2024 Feb 28;25(5):2790. doi: 10.3390/ijms25052790 (PMC10932171; doi:10.3390/ijms25052790)
Supplement: Supplementary file 1 [file ijms-25-02790-s001.zip › ijms-2841513-SI.pdf]

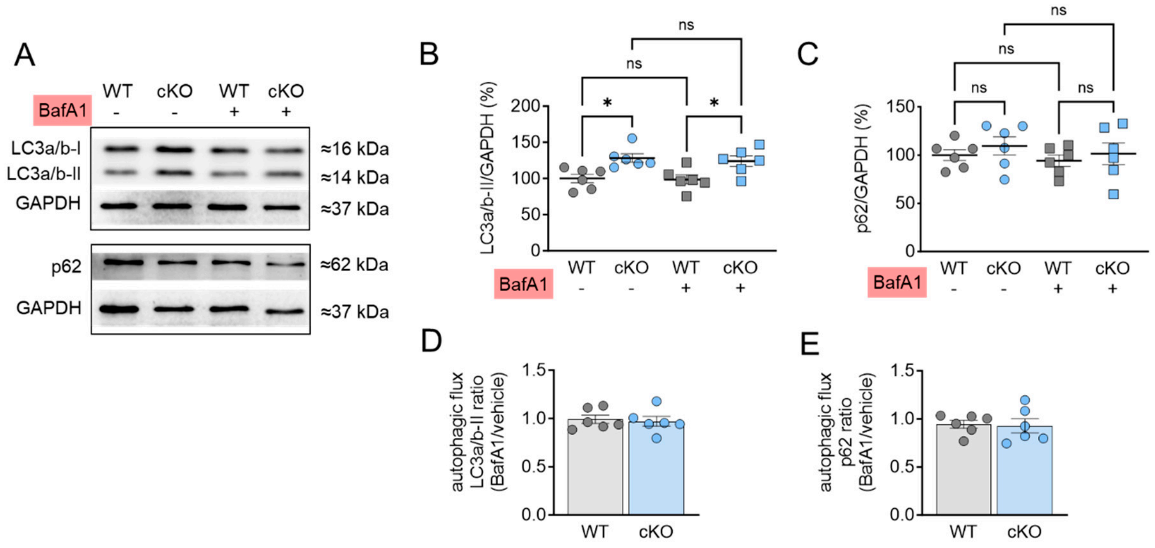

Suppl. Figure S1: ***Ex vivo*** **Bafilomycin (BafA1) treatment PKD1 cKO versus WT. (A)** Representative Western blots of LC3-**I** and **II** (above) and p62 (below) and the corresponding loading controls GAPDH in the absence (-) and presence (+) of the inhibitor Bafilomycin A1 (BafA1). Levels of **(B)** LC3-**II** over GAPDH and **(C)** p62 over GAPDH in the absence (-) and presence (+) of BafA1. Autophagic flux ratios of (**D**) LC3-**II** and **(E)** p62. Data are shown as mean ± SEM; n=6 samples/group. * $p < 0.05$ PKD1cKO vs. WT ; before vs. after BAfA1 treatment by one-way ANOVA. P-values were corrected for multiple comparisons by the Tukey method.
